# *Mycobacterium avium* infection induces the resistance of the interferon-γ response in mouse spleen cells at late stages of infection

**DOI:** 10.1186/s41232-016-0024-3

**Published:** 2016-08-26

**Authors:** Atsuko Masumi, Keiko Mochida, Kazuya Takizawa, Takuo Mizukami, Madoka Kuramitsu, Momoka Tsuruhara, Shigetarou Mori, Keigo Shibayama, Kazunari Yamaguchi, Isao Hamaguchi

**Affiliations:** 1 Department of Safety Research on Blood and Biological Products, National Institute of Infectious Diseases, Tokyo, Japan; 2 Department of Bacteriology II, National Institute of Infectious Diseases, 4-7-1, Gakuen Musashimurayama-shi, Tokyo, 208-0011 Japan; 3 Present address: Faculty of Pharmaceutical Sciences, Aomori University, 2-3-1, Kohbata, Aomori-shi, Aomori, 030-0943 Japan

**Keywords:** *Mycobacterium avium*, Infection, Hematopoietic stem cells, Bone marrow, Spleen, Interferon regulatory factor, IFN-γ

## Abstract

**Background:**

Bacterial infections cause an increase in the population of hematopoietic stem cells (HSCs). To investigate the downstream factors associated with hematopoietic stem cells, mice are infected with *Mycobacterium avium* (*M. avium*).

**Results:**

*Mycobacterium avium* (*M. avium*) infection induces the enlargement of the spleen and changes in histopathology, including changes to the lineage populations. A dramatic expansion of Lin^−^c-kit^+^Sca-1^+^ (KSL) cells in mouse bone marrow cells and spleen cells was detected 4 weeks after infection with *M. avium*; however, there was no difference in the engraft activity between infected and un-infected mouse bone marrow cells. We tested the cytokine and cytokine-related gene expression after *M. avium* infection and found that IFN-γ expression increased and peaked at 4 weeks in both bone marrow and spleen cells. The expression of Sca-1 gene peaked at 4 weeks in the bone marrow but peaked at 2 weeks in spleen cells, although the Sca-1 surface marker peaked at 4 weeks after infection in both bone marrow and spleen cells. Interferon regulatory factor-2 (IRF-2) expression did not change in the bone marrow cells, whereas it decreased in spleen cells at 4 weeks and IRF-1 expression was up-regulated in both bone marrow and spleen cells after infection. However, the up-regulation of IRF-1 was not correlated with IFN-γ expression in the *M. avium*-infected mouse spleen cells.

**Conclusions:**

This finding suggests that the IFN-γ production mediated by *M. avium* infection alters the population of KSL cells during host defense, and the down-regulation of the IFN-γ response in spleen cells occurs at the late stage after *M. avium* infection.

## Background

Hematopoietic stem cells (HSCs) are functionally defined by their unique capacity for self-renewal and differentiation into all types of mature blood cells [1]. Under normal conditions, the process of hematopoietic stem cell self-renewal and their conversion into lineage-committed progenitors is tightly controlled to maintain blood cell production [1, 2]. It has been shown that the equilibrium of bone marrow hematopoiesis is altered during bacterial infection, whereby the production of phagocytes, particularly granulocytes and monocytes, becomes predominant with the concomitant inhibition of the development of other lineages. Despite the biological significance of changes in hematopoiesis during bacterial infection, relatively, little is known about the downstream factors associated with hematopoietic precursor cells, including hematopoietic stem cells.

During bacterial infection, the bone marrow hematopoietic activity shifts toward granulocyte production, and granulocyte colony-stimulating factor (G-CSF) and chemokines, which are critical for host defenses, are produced [3]. Bone marrow (BM) hematopoietic stem and progenitor cells (HSPCs) can be activated by type I IFNs, viruses, and bacterial infection to increase the level of hematopoiesis. The dramatic expansion of the lin-c-kit^+^Sca-1^+^ cell pool is induced by bacterial infection due to IFN-γ production [4].

Following intraperitoneal inoculation of C57BL/6, DBA/1, and BALB/c mice with *Mycobacterium avium* (*M. avium*), bacillary growth in the liver, spleen, and peritoneal cavity was observed [5]. *M. avium* infection seems to induce an increase in the proliferative fraction of primitive LT-HSCs (long-term HSCs), along with a substantial increase in the number of early-committed progenitors. *M. avium* is an opportunistic pathogen that commonly infects immunodepressed patients and lives inside macrophage phagosomes [6]. The importance of *M. avium* has been increasing due to the high incidence of infections with this pathogen in immunocompromised patients, namely those with AIDS. IFN-*γ* is strongly up-regulated during *M. avium* infection [7]. Previously, we reported that interferon regulatory factor-2 was up-regulated and induced megakaryopoiesis in hematopoietic stem/progenitor cells in an inflammation state, such as under enhanced IFN-γ production [8]. IFN-γ is a critical defense against mycobacteria, as mice with a disrupted IFN-γ receptor gene, IFNGR-1, did not respond to *M. avium* infection [9], and human patients with mutations in the IFN-γ receptor died with disseminated *M. avium* infection [10].

In the present study, we analyzed Interferon regulatory factor (IRF)-1, IRF-2, and IFN-γ expression in mouse bone marrow and spleen cells after *M. avium* infection. Up-regulated by *M. avium* infection, IFN-γ induces the expression of IFN-γ-responsive genes; however, continuous infection down-regulates IFN-γ responsiveness at the late stage and suggests chronic infection.

## Methods

### Mouse infection

C57BL/6 female mice were infected with *M. avium* strain 25291 (10^6^ cfu) (obtained from ATCC) intravenously. The mice were sacrificed at 24 h, 2 weeks, 1 month, or 3 months after infection, and the lungs, BM, and spleen were harvested for further analysis. All protocols were approved by the Institutional Animal Care and Use Committees of National Institute of Infectious Diseases.

### Antibodies and cell purification

Rat IgG2b anti-mouse lineage markers conjugated to PE, APC-c-kit, FITC-Sca1, APC-CD45.2, and PE-CD45.1 were purchased from eBioscience (San Diego, CA, USA). Mononuclear cells from bone marrow and spleen populations were isolated from *M. avium*-infected mice, and the low-density cells were isolated using Histopaque gradients (Sigma, St Louis, MO, USA). The cells were stained with an antibody cocktail consisting of PE-conjugated anti-Gr-1, Mac-1, B220, CD4, and CD8 monoclonal antibodies (BD Biosciences). Lineage-positive cells were depleted using Anti-PE MicroBeads (Miltenyi Biotec), and the lineage-depleted cells were further stained with FITC-Sca-1 and APC5-c-kit antibodies to isolate the lin^−^c-kit + sca-1+ (KSL) cells. Fluorescence analysis and sorting were performed using a flow cytometry (JSAN Bay Bioscience, Kobe, Japan).

### Quantitative real-time RT-PCR

Real-time RT-PCR was performed as previously described [6]. Total RNA was isolated from 1 × 10^4^ to 1 × 10^5^ bone marrow cells using the ISOGEN reagent (Toyobo, Japan) according to the manufacturer’s instructions (Nippongene, Tokyo, Japan), and the cDNA was reverse transcribed using Superscript III (Invitrogen). The expression level of specific mRNAs was analyzed by quantitative RT-PCR using a LightCycler instrument (Roche Diagnostics), and the PCR was performed using SYBR PREMIX Ex-Taq according to the manufacturer’s instructions (Takara, Japan). The data are presented as expression levels (2^−ΔCt^ compared with either GAPDH or β-actin as the control). The mouse IRF-1 and IRF-2 primers have been previously reported [6]. The mouse Sca-1 primers are as follows: gtttgctgattcttcttgtggccc and actgctgcctcctgagtaacacac.

### Histologic preparation and periodic acid-Schiff-hematoxylin staining

*M. avium*-infected or *M. avium-*un-infected mouse spleen was harvested and fixed in Bouin solution (Sigma-Aldrich) or 4 % (*w*/*v*) paraformaldehyde in phosphate-buffered saline (pH 7.5) at 4 °C for 24 h. After fixation, samples were dehydrated in a graded ethanol series and cleared in xylene and then embedded in paraffin; 4-μm semithin sections were prepared using a carbon steel blade (Feather Safety Razor Co) by microtome (Yamato Kouki). Tissue sections were mounted on super-frosted glass slides coated with methyl-amino-silane (Matsunami Glass) and dried overnight and stained with hematoxylin and eosin (HE) and periodic acid-Schiff (PAS). Cellular polysaccharide deposits were detected using the PAS reaction.

Histologic images were acquired using a NikonEclipse E1000 microscope equipped with ×10/0.30, ×20/0.50, ×40/0.75, and ×100/1.30 NA objective lenses. Images were captured with a Nikon DXM 1200F digital camera.

### Transplantation

Bone marrow cells were isolated from *M. avium*-infected mice (C57BL/6-Ly5.2), and 2 × 10^6^ whole bone marrow mononuclear cells were injected into X-ray irradiated (9.5 Gy) C57BL/6-Ly5.1 mice. At five, 9, 13, and 17 weeks after transplantation, the isolated peripheral blood from the recipient mice was stained with PE-CD45.1 and FITC-CD45.2 antibodies, and chimerism was analyzed. Donor chimerism was determined as %CD45.2^+^/%CD45.1^+^ + %CD45.2^+^.

## Results

**Fig. 1:**
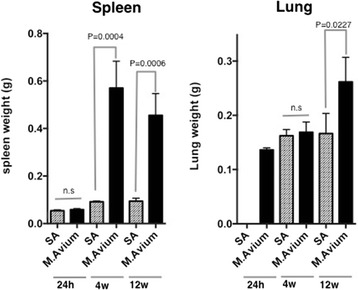
Spleen and lung weight of mice infected with *M. avium.* C57BL/6 female mice were infected with *M. avium* strain 25291(10^6^ cfu) intravenously and the mice were sacrificed at 24 h and 2, 4, and 12 weeks, and the weight of spleen and lungs were analyzed. Weights of lung 24 h after SA injection were not detected. *n.s* indicates no significant. Each value is presented as mean ± standard deviation (*n* = 5). Significant differences (*P* < 0.05) were determined using Student’s *t* test

**Fig. 2:**
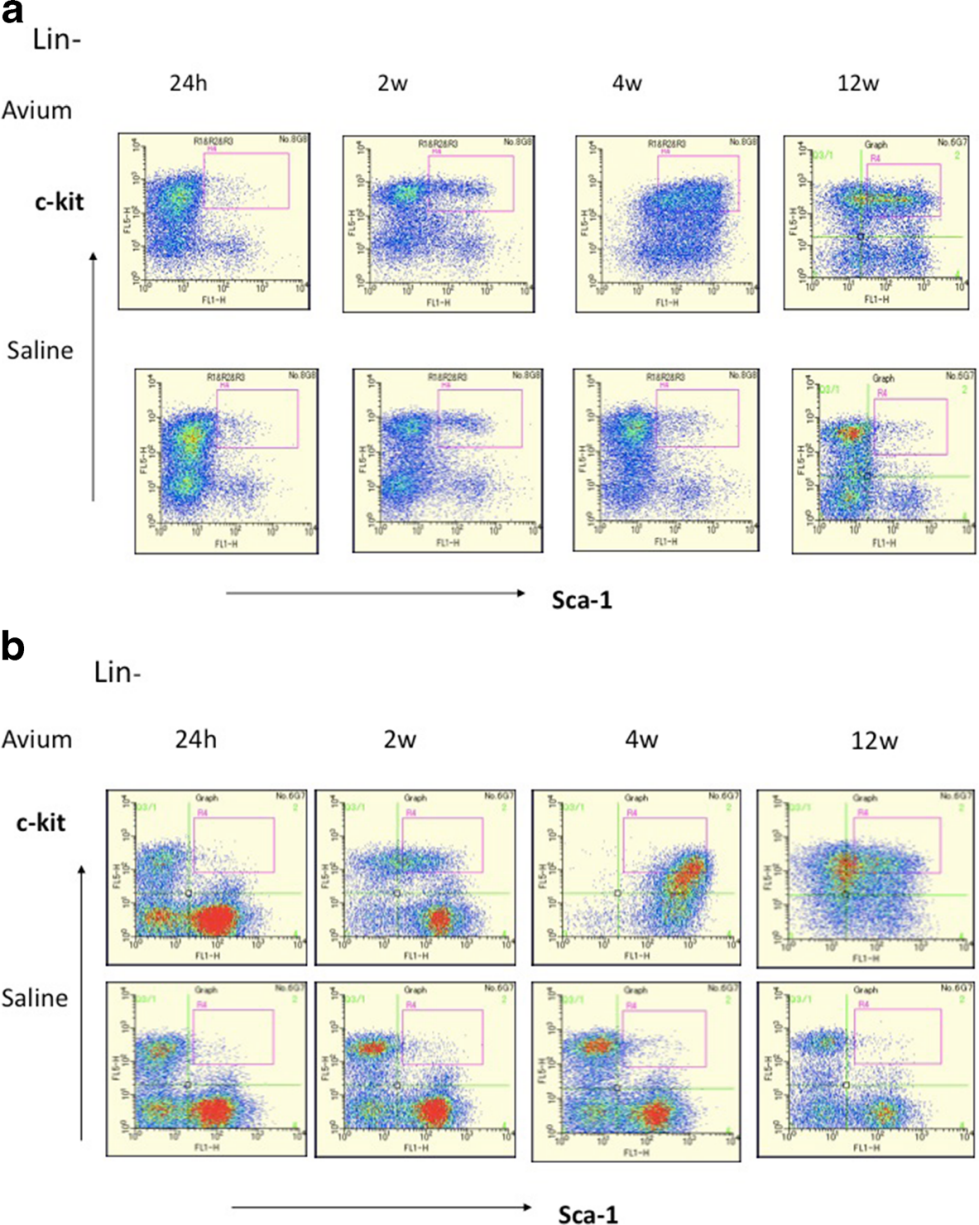
FACS analysis for bone marrow and spleen from mice infected *M. avium*. Mice infected with *M. avium* strain 25291 were sacrificed at 24 h and 2, 4, and 12 weeks, and mononuclear cells from bone marrow and spleen populations were isolated. The lin-c-kit^+^Sca-1^+^cell population in the bone marrow cells (**a**) and spleen cells (**b**) were isolated. Data are representative of two experiments with similar results

When injected intravenously with *M. avium*, mice were systemically infected with a large number of bacteria 4 weeks after injection (data not shown), and the sizes of their spleens increased by over sixfold at 4 weeks after consistent infection (Fig. 1). The sizes of their lungs increased significantly at 12 weeks after *M. avium* infection. To examine the changes in the bone marrow hematopoietic precursor cell populations following the systemic infection with *M. avium*, the bone marrow cells were analyzed by flow cytometry on the basis of their hematopoietic surface markers. As shown in Fig. 2, the lin-c-kit + Sca-1+ cell population in the bone marrow of the control mice was very small, whereas the number of lin-c-kit + Sca-1+ cells in the bone marrow was markedly increased in mice infected with *M. avium*. At 24 h post-i.v. *M. avium* treatment, the size of the KSL cell population did not significantly increase in the bone marrow; however, the KSL population had increased by 2 weeks and reached a maximum size after 4 weeks post-i.v.; this population was decreased at 12 weeks after injection (Fig. 2a). We also analyzed spleen cells from mice infected with *M. avium*. The KSL population of spleen cells increased by 2 weeks, reached a maximum by 4 weeks, and then had decreased at 12 weeks after infection, results that are similar to those for the bone marrow cells (Fig. 2b). We found that *M. avium* infection dramatically modulated the KSL populations of both the bone marrow and the spleen.

**Fig. 3:**
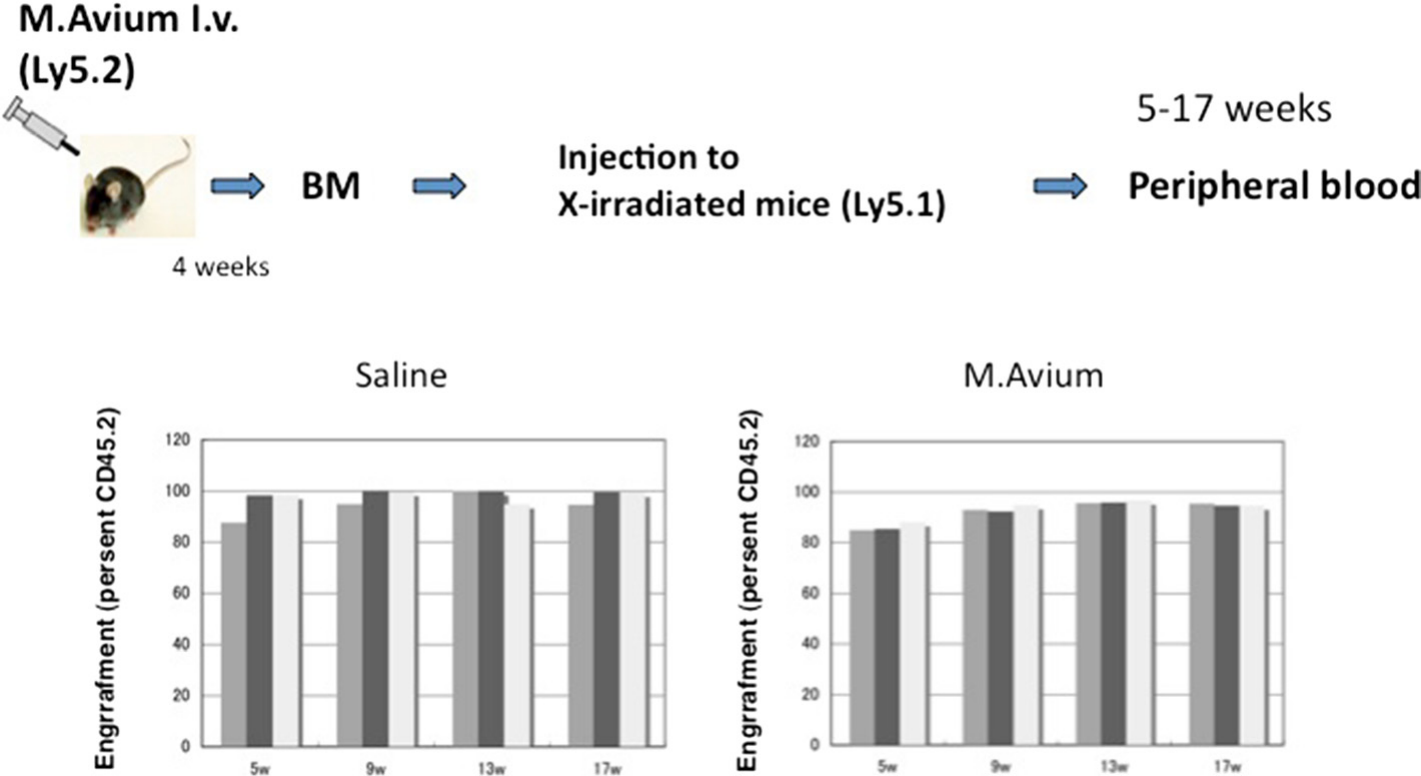
Transplantation. Bone marrow cells were isolated from *M. avium*-infected mice and 2 × 10^6^ whole bone marrow mononuclear cells were injected into X-ray irradiated mice (*n* = 3 recipients). At 5, 9, 13, and 17 weeks after transplantation, the isolated peripheral blood from the recipient mice were stained and chimerism were analyzed

To analyze the functional properties of hematopoietic stem cells (HSCs) from *M. avium*-infected mice, we isolated the bone marrow cells from mice infected with *M. avium* for 4 weeks and performed noncompetitive transplant assays. Five, 9, 13, and 17 weeks after the injection of the bone marrow cells, the chimerism of peripheral blood cells was analyzed. The transplantation of 2 × 10^6^ bone marrow cells from infected mice or health controls is normally sufficient to rescue and fully populate the hematopoietic system of all lethally irradiated recipients (Fig. 3). Despite the great alteration of the bone marrow KSL population, the engraftment efficiency was comparative to the control.

**Fig. 4:**
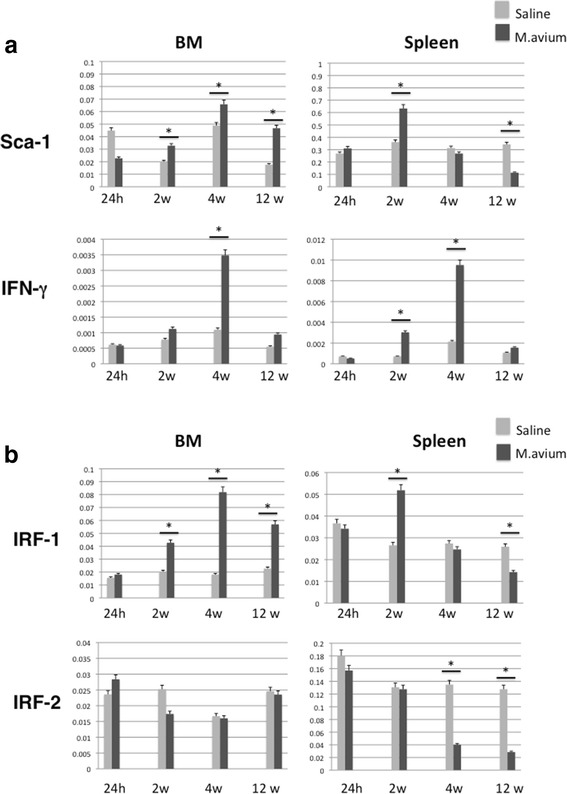
Real-time PCR analysis. Bone marrow and spleen cells were isolated from mice at 24 h and 2, 4, and 12 weeks after infection and mRNA of Sca-1 and IFN-γ (**a**) and IRF-1 and IRF-2 (**b**) were analyzed. Data represent two independent experiments each performed in triplicate. Each value is presented as mean ± standard deviation. Significant differences (*P < 0.05) were determined using Student’s *t* test

According to our FACS analysis, the significant increase in the KSL population was due to the induction of the Sca-1 surface marker. We analyzed Sca-1 mRNA expression in the bone marrow and spleen cells of mice infected with *M. avium*, and the real-time PCR analysis showed that the Sca-1 expression in the bone marrow cells had significantly increased at 12 weeks after infection. The Sca-1 expression in the spleen cells was increased at 2 weeks after infection and decreased thereafter until 12 weeks after infection (Fig. 4a). Sca-1 expression is reported to be regulated by IFN stimulation; thus, we investigated IFN expression after infection with *M. avium*. The expression of IFN-γ was enhanced at 2 weeks, reached a maximum at 4 weeks after infection and then decreased in both bone marrow and spleen cells (Fig. 4a). We detected a three- to fivefold induction in the infected cells relative to the level in the saline controls. In contrast, the type I IFN (IFN-α and IFN-β) expression level did not change after infection in either the bone marrow or spleen cells (data not shown). Interferon regulatory factor, IRF-1, also was up-regulated at 2 weeks, reached a maximum expression level at 4 weeks, and was reduced by 12 weeks after infection, results that correlated with the expression of IFN-γ (Fig. 4b). IRF-1 expression in the spleen cells was enhanced at 2 weeks post-i.v. and decreased by 4 and 12 weeks after infection (Fig.4b). We found no change in the expression level of IRF-2 in the bone marrow cells. IRF-2 expression was decreased at 4 and 12 weeks after infection in comparison with the saline controls (Fig. 4b). In the spleen cells, the expression level of Sca-1 was increased at 2 weeks post-i.v. and decreased by 4 and 12 weeks after infection, results that are similar to those for the IRF-1 expression in the spleen cells.

**Fig. 5:**
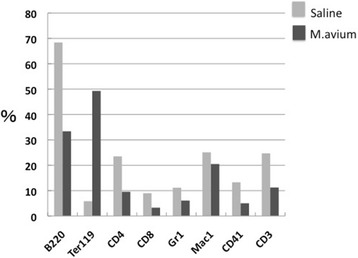
Lineage analysis for spleen from mice infected *M. avium*. The spleen cells from mice infected with *M. avium* for 4 weeks were stained with indicated lineage markers. Data are representative of two independent experiments with similar results

We observed splenomegaly in the mice after infection with *M. avium* (Fig. 1). To examine the lineage populations in the spleen cells from mice infected with *M. avium* for 4 weeks, the spleen cells were stained with PE-conjugated lineage markers and analyzed by flow cytometry. As shown in Fig. 5, the B and T cell lineage markers were decreased compared with the control, and Ter119, an erythroid-specific marker, was dramatically enhanced. Gr-1- and CD41-positive cells were also reduced in the spleens from *M. avium*-infected mice. As shown in Fig. 4b, the IRF-2 expression was reduced at 4 and 12 weeks after *M. avium* infection in the spleen cells, a result that may be associated with the reduction of lineage markers. *M. avium* infection increases a proportion of Ter119-positive cells and reduces other lineage cells, including B220.

**Fig. 6:**
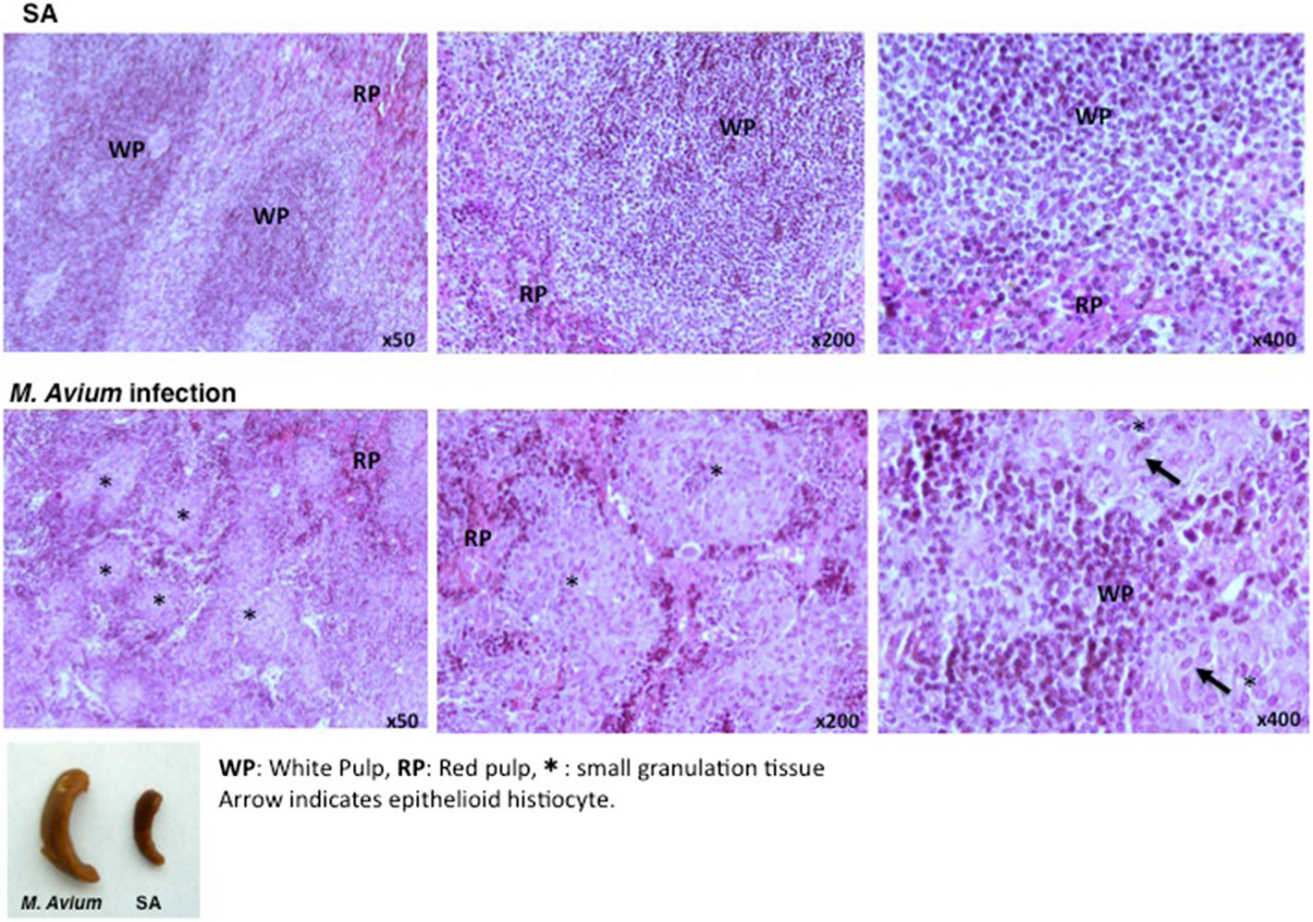
Histopathology of spleen after 7 weeks *M. avium* infection. Mouse spleen after 7 weeks *M. avium* infection were stained with HE and PAS. *WP* indicates white pulp. *RP* indicates red pulp. *Asterisk* indicates small granulation tissue. *Arrow* indicates epithelioid histiocyte. Mice spleen after 7 weeks *M. avium* infection and saline (SA) injection were indicated in the *left bottom*. Data are representative of two independent experiments with similar results

In spleen cells, the IFN-γ expression level was up-regulated at 4 weeks after *M. avium* infection; however, the expression levels of IRF-1-, IRF-2- and Sca-1-, and IFN-γ-responsive genes did not increase or decrease until 4–12 weeks after infection. The histopathology of the spleen at 7 weeks after *M. avium* infection is shown in Fig. 6. The white pulp had disappeared, and small granules and epithelioid histiocytes appeared in the spleens from the infected mice, which may have led to the relative reduction of IRF and Sca-1 expression in the splenocytes. The red pulp area was enlarged in the infected spleens, reflecting the increase in the number of the Ter119-positive cells in the spleen (Figs. 5 and 6).

## Discussion and conclusions

We demonstrated that *M. avium* infection induced the expression of IFN-γ inducible genes at an early stage but not at late stages in spleen cells. In the bone marrow cells, the expression levels of these IFN-γ-inducible genes increased in parallel with IFN-γ expression. *M. avium* infection induced the dramatic expansion of KSL cells in the bone marrow, and no significant difference in the engraftment activity in the bone marrow of infected and un-infected mice was detected. According to Baldridge et al., IFN-γ stimulation reduced LT-HSC and engraftment efficiency in bone marrow [7]. Less amount of whole bone marrow transplantation might show the reduction of engraftment activity, but 1 × 10^6^ cells of whole bone marrow engraftment was not detected the notable difference between infected and un-infected mice. In fact, 5 weeks after transplantation, HSC from *M. avium*-infected mice showed slightly impaired engraftment compared to that of saline control. Stat1 and IRF-1 are involved in downstream of IFN-γ. Aly et al. reported that Stat-1-KO mice and IRF-1-KO mice had higher levels of *M. avium* in their lungs than wild-type mice but did not develop granuloma necrosis [11]. They investigated the effect of *M. avium* infection for IFN-dependent genes and concluded that IRF-1 might be regulator of mycobacteria-induced immunopathology [11]. In our study, IRF-1 expression increased concomitant with the change in IFN-γ expression at 4 weeks after *M. avium* infection in bone marrow. IRF-1 expression was dramatically enhanced by IFN-γ stimulation in many types of cells. In contrast, IRF-2 expression did not change in mouse bone marrow cells after *M. avium* infection. In a previous study, we demonstrated that IRF-2 expression was up-regulated in mouse hematopoietic stem/progenitor cells by IFN-γ stimulation in vitro [8], and IRF-2 up-regulation by IFN-γ stimulation may be specific for hematopoietic stem/progenitor cells in bone marrow cells. In spleen cells, IRF-1 expression was greater at 2 weeks after infection. In fact, IFN-γ expression was significantly enhanced 2 weeks after infection. However, after infection, the expression of IRF-1 was not greater at 4 weeks when the maximum level of IFN-γ expression level was attained in spleen cells. In parallel with the IRF-1 expression in the spleen cells, Sca-1 expression was enhanced at 2 weeks but not at 4 weeks after infection. The expression levels of both IRF-1 and Sca-1 were significantly decreased compared with the saline controls at 12 weeks after infection. Thus, in mouse bone marrow cells, IRF-1 and Sca-1 expression was concomitant with that of IFN-γ, but their expression was independent of IFN-γ signaling in spleen cells. In the spleen cells of infected mice, IRF-2 expression was not enhanced at any time and was reduced at 4 and 12 weeks after infection relative to the saline control mice. The expression of both IRF-1 and IRF-2 may be regulated by secondary factors that are mediated by *M. avium* infection in spleen cells in vivo.

Gomes-Pereira et al. reported that mice of two genetically determined iron-overload phenotypes, Hfe−/− and b2m−/−, show an increased susceptibility to experimental infection with *M. avium* and that, during infection, these animals accumulate iron inside granuloma macrophages [12]. Lafuse et al. investigated whether mycobacteria-infected macrophages are poor responders to interferon-γ (IFN-γ), resulting in the decreased expression of IFN-γ-induced genes [13]. They demonstrated that Mycobacteria inhibit IFN-γ-induced gene expression by multiple pathways that involve both TLR2 and non-TLR receptors. Furthermore, production of TGF-β by *M. avium* infection to macrophage is reported to be associated with unresponsiveness to IFN-γ [14]. In our study, *M. avium* infection-induced IFN-γ expression resulted in the expression of IFN-γ-inducible genes at an early stage; however, at a late stage after infection, the dramatic induction of IFN-γ did not increase, and rather reduced, the expression levels of these genes. The FACS analysis of the spleen KSL cells showed a greatly enhanced Sca-1-positive population despite the unchanged or decreased Sca-1 gene expression at 4 and 12 weeks after infection. At the late stage of *M. avium* infection, the IFN-γ responsiveness was down-regulated in the spleen cells, resulting in the disruption of the regulation of the host defenses.

Upon vaccinia virus (VV) infection, both the proportion and number of KSL cells were dramatically increased as early as 1 day after infection, and the KSL population returned to normal at 7 days postinfection [15]. A similar increase was observed in Lin-c-kit^+^sca-1^+^ in *Escherichia coli*-infected Balb/c mice, whereas the Lin-c-kit^+^sca-1- cell population was decreased dramatically during the early period of infection [4]. In contrast, enhanced KSL cell population in mice infected with *M. avium* was detected at 2–4 weeks and decreased at 12 weeks after infection. Chronic IFN-γ exposure causes anemia because IRF-1/PU-1 induction by IFN-γ exposure induces impaired erythropoiesis [16]. As shown in Figs. 5 and 6, *M. avium* infection induced splenomegaly with ter119-positive cells and enlargement red pulp. The IRF-2 expression did not change in the bone marrow cells during *M. avium* infection and was decreased at 4 and 12 weeks after infection in the spleen cells. This reduction of IRF-2 expression in the spleen cells may due to the dramatic induction of Ter119-positive cells and the reduction of other cell lineages (Fig. 5). We showed that IRF-2 expression was relatively high in hematopoietic stem cells (CD34-KSL) [8], and we speculate that the increase of Ter119-positive cells in the spleen relatively reduces the expression of IRF-2. Baldridge et al. showed LT-HSCs are highly proliferative during chronic *M. avium* infection in the bone marrow and mobilize to leave the bone marrow to spleen [7]. Impaired LT-HSCs are possible to mobilize to spleen from bone marrow and affect the lineage population in spleen cells. However, *M. avium* infection increases a proportion of Ter119-positive cells and reduced other lineage cells, resulted in the splenic impaired hematopoietic and immune system. Induction of IFN-γ at early stage of *M. avium* infection cannot continue to improve host defense due to the splenic impaired hematopoiesis. Our results could lead to improved clinical aspects for pathophysiology of both hematopoietic and immune system in patients with chronic infections, such as tuberculosis and AIDS. Further investigation is required regarding the mechanism of the association of *M. avium* infection with cytokine and cytokine-related genes in hematopoietic stem cells.

## Abbreviations

HSCs: Hematopoietic stem cells
*M. avium*: *Mycobacterium avium*
KSL: Lin^−^c-kit^+^Sca-1^+^
LT-HSCs: Long-term HSCs
IRF: Interferon regulatory factor
G-CSF: Colony-stimulating factor
BM: Bone marrow
HSPCs: Hematopoietic stem and progenitor cells
HE: Hematoxylin and eosin
PAS: Periodic acid-Schiff

## References

[1] Kondo M, Wagers A, MG M (2003). Biology of hematopoietic stem cells and progenitors: implications for clinical application. Annu Rev Immunol.

[2] Akala O, Clarke M (2006). Hematopoietic stem cell self-renewal. Curr Opin Genet Dev.

[3] Pauksen K, Elfman L, Ulfgren AK, Venge P (1994). Serum levels of granulocyte-colony stimulating factor (G-CSF) in bacterial and viral infections, and in atypical pneumonia. Br J Haematol.

[4] Zhang P, Nelson S, Bagby GJ, Siggins R, Shellito JE, Welsh DA (2008). The lineage-c-Kit + Sca-1+ cell response to Escherichia coli bacteremia in Balb/c mice. Stem Cells.

[5] Appelberg R, Sarmento A (1990). The role of macrophage activation and Bcg-encoded macrophage functions in the control of mycobacterium avium infection in mice. Clin Exp Immunol.

[6] Wagner D, Maser J, Lai B, Cai Z, Barry III CE, Honer zu Bentrup K, Russell DG, Bermudez LE. Elemental analysis of Mycobacterium avium-, Mycobacterium tuberculosis-, and mycobacterium smegmatis-containing phagosomes indicates pathogen-induced microenviroments within the host cell's endosomal system. J. Immunol. 2005;174:1491–1500. 10.4049/jimmunol.174.3.1491 15661908

[7] Baldridge MT, King KY, Boles NC, Weksberg DC, Goodell MA (2010). Quiescent haematopoietic stem cells are activated by IFN-gamma in response to chronic infection. Nature.

[8] Masumi A, Hamaguchi I, Kuramitsu M, Mizukami T, Takizawa K, Momose H, Naito S, Yamaguchi K. Interferon regulatory factor-2 induces megakaryopoiesis in mouse bone marrow hematopoietic cells. FEBS Lett. 2009;583:3493–500. 10.1016/j.febslet.2009.10.006 19818776

[9] Feng C, Weksberg D, Taylor G, Sher A, Goodell M (2008). The p47 GTPase Lrg-47 (Irgm1) links host defense and hematopoietic stem cell proliferation. Cell Stem Cell.

[10] Horwiz ME, Uzel G, Linton GF, Miller JA, Brown MR, Malech HL, Holland SM. Persistent Mycobacterium avium infection following nonmyloablative allogenic peripheral blood stem cell transplantation for interferon-g receptor-1 deficiency. Blood. 2003;102:2692–4. 10.1182/blood-2003-04-1268 12805054

[11] Aly S, Mages J, Reiling N, Kalinke U, Decker T, Lang R, Ehlers S. Mycobacteria-induced granuloma necrosis depends on IRF-1. J Cell Mol Med. 2009;13:2069–82. 10.1111/j.1582-4934.2008.00470.x PMC6512360 18705699

[12] Gomes-Pereira S, Rodrigues P, Appelberg R, Gomes M (2008). Increased susceptibility to Mycobacterium avium in hemochromatosis protein HFE-deficient mice. Infect Immun.

[13] Lafuse WP, Alvarez GR, Curry HM, Zwilling BS (2006). Mycobacterium tuberculosis and Mycobacterium avium inhibit IFN-gamma-induced gene expression by TLR2-dependent and independent pathways. J Interferon Cytokine Res.

[14] Bermudez LE (1993). Production of transforming growth factor-beta by Mycobacterium avium-infected human macrophages is associated with unresponsiveness to IFN-gamma. J Immunol.

[15] Singh P, Yao Y, Weliver A, Broxmeyer HE, Hong S-C, Chang C-H. Vaccinia virus infection modulates the hematopoietic cell compartments in the bone marrow. Stem Cells. 2008;26:1009–1016. 10.1634/stemcells.2007-0461 PMC2814369 18258722

[16] Libregts SF, Gutierrez L, de Bruin AM, Wensveen FM, Papadopoulos P, van Ijcken W, Ozgur Z, Philipsen S, Nolte MA (2011). Chronic IFN-gamma production in mice induces anemia by reducing erythrocyte life span and inhibiting erythropoiesis through an IRF-1/PU.1 axis. Blood.

